# Automated MRI liver segmentation for anatomical segmentation, liver volumetry, and the extraction of radiomics

**DOI:** 10.1007/s00330-023-10495-5

**Published:** 2024-01-13

**Authors:** Moritz Gross, Steffen Huber, Sandeep Arora, Tal Ze’evi, Stefan P. Haider, Ahmet S. Kucukkaya, Simon Iseke, Tom Niklas Kuhn, Bernhard Gebauer, Florian Michallek, Marc Dewey, Valérie Vilgrain, Riccardo Sartoris, Maxime Ronot, Ariel Jaffe, Mario Strazzabosco, Julius Chapiro, John A. Onofrey

**Affiliations:** 1 Department of Radiology and Biomedical Imaging, Yale University School of Medicine, New Haven, CT USA; 2 Charité Center for Diagnostic and Interventional Radiology, Charité - Universitätsmedizin Berlin, Berlin, Germany; 3 Department of Biomedical Engineering, Yale University, New Haven, CT USA; 4 Department of Otorhinolaryngology, University Hospital of Ludwig Maximilians Universität München, Munich, Germany; 5 Department of Diagnostic and Interventional Radiology, Pediatric Radiology and Neuroradiology, Rostock University Medical Center, Rostock, Germany; 6 Department of Diagnostic and Interventional Radiology, University Duesseldorf, Duesseldorf, Germany; 7 Université Paris Cité, Île-de-France, Paris, France; 8 Department of Radiology, Hôpital Beaujon, AP-HP.Nord, Department of Radiology, Île-de-France, Clichy, France; 9 Department of Internal Medicine, Yale University School of Medicine, New Haven, CT USA; 10 Department of Urology, Yale University School of Medicine, New Haven, CT USA

**Keywords:** Magnetic resonance imaging, Liver, Computer-assisted image analyses, Deep learning

## Abstract

**Objectives:**

To develop and evaluate a deep convolutional neural network (DCNN) for automated liver segmentation, volumetry, and radiomic feature extraction on contrast-enhanced portal venous phase magnetic resonance imaging (MRI).

**Materials and methods:**

This retrospective study included hepatocellular carcinoma patients from an institutional database with portal venous MRI. After manual segmentation, the data was randomly split into independent training, validation, and internal testing sets. From a collaborating institution, de-identified scans were used for external testing. The public LiverHccSeg dataset was used for further external validation. A 3D DCNN was trained to automatically segment the liver. Segmentation accuracy was quantified by the Dice similarity coefficient (DSC) with respect to manual segmentation. A Mann-Whitney *U* test was used to compare the internal and external test sets. Agreement of volumetry and radiomic features was assessed using the intraclass correlation coefficient (ICC).

**Results:**

In total, 470 patients met the inclusion criteria (63.9±8.2 years; 376 males) and 20 patients were used for external validation (41±12 years; 13 males). DSC segmentation accuracy of the DCNN was similarly high between the internal (0.97±0.01) and external (0.96±0.03) test sets (*p*=0.28) and demonstrated robust segmentation performance on public testing (0.93±0.03). Agreement of liver volumetry was satisfactory in the internal (ICC, 0.99), external (ICC, 0.97), and public (ICC, 0.85) test sets. Radiomic features demonstrated excellent agreement in the internal (mean ICC, 0.98±0.04), external (mean ICC, 0.94±0.10), and public (mean ICC, 0.91±0.09) datasets.

**Conclusion:**

Automated liver segmentation yields robust and generalizable segmentation performance on MRI data and can be used for volumetry and radiomic feature extraction.

**Clinical relevance statement:**

Liver volumetry, anatomic localization, and extraction of quantitative imaging biomarkers require accurate segmentation, but manual segmentation is time-consuming. A deep convolutional neural network demonstrates fast and accurate segmentation performance on T1-weighted portal venous MRI.

**Key Points:**

*• This deep convolutional neural network yields robust and generalizable liver segmentation performance on internal, external, and public testing data.*

*• Automated liver volumetry demonstrated excellent agreement with manual volumetry.*

*• Automated liver segmentations can be used for robust and reproducible radiomic feature extraction.*

**Supplementary Information:**

The online version contains supplementary material available at 10.1007/s00330-023-10495-5.

## Introduction

Magnetic resonance (MR) imaging (MRI) offers high tissue contrast and can be used for non-invasive assessment of the liver, and is integral to diagnosing hepatocellular carcinoma (HCC) [1], liver fibrosis [2], cirrhosis [3], and portal hypertension [4]. Liver segmentation can be used for volumetry, anatomic localization, and the extraction of radiomics. Accurate liver volumetry is essential for risk assessment, decision management, and planning of therapeutic procedures. An important predictor of the success of liver transplantation is the liver volume for both donor and recipient [5]. Liver resection also relies on reliable volume measurements as the outcome is heavily dependent on the liver remnant [6]. To plan therapy and calculate dosimetry, liver volumetry is important for Yttrium-90 selective internal radiotherapy [7] and liver volumes are also of interest for epidemiology research [8]. Anatomic localization by accurate liver segmentation is key for anatomical guidance in computer-assisted surgery [9] and radiotherapy [8, 10], and is accomplished through manual segmentation. Moreover, liver segmentation is a pivotal pre-processing step for lesion detection algorithms [11] and for the extraction of radiomics.

Manual liver segmentation is time-consuming and subject to inter-rater variation [12], which limits its practicality in clinical practice workflows. Convolutional neural networks (CNN) based on deep learning have shown promising results in automating segmentation tasks in medical imaging [13] and provide fast processing times. However, overfitting and dataset shift are major problems in deep learning and external evaluation is pivotal to ensure generalizable validity [14] and many deep learning algorithms have shown substantially decreased performance on external data [15]. A recent study underlined the importance of model evaluation on datasets composed of heterogeneous diagnostic findings encountered in clinical practice [16]. Most proposed automated liver segmentation methods were developed on small datasets and tested only on small internal test sets and therefore do not guarantee generalizable and consistent performance on data from other institutions [14, 15]. Liver image analysis techniques based on radiomics and deep learning, which rely on anatomical segmentations as input, have demonstrated their utility in applications such as characterizing focal hepatic lesions, staging liver fibrosis, and identifying portal hypertension [17].

The aim of this study was to develop and evaluate a deep CNN (DCNN) for automated liver segmentation, liver volumetry, and radiomic feature extraction on portal venous phase contrast-enhanced MRI using a large institutional dataset and assess performance generalizability to external and public testing data.

## Materials and methods

### Compliance with ethical standards

This retrospective study is HIPPA-compliant and was approved by the institutional review boards of the Yale School of Medicine and the Beaujon Hospital in Paris with full waiver of informed consent and was conducted in accordance with the Declaration of Helsinki.

### Data availability

Image data used in this paper cannot be shared publicly due to legal reasons (it would compromise patient confidentiality).

### Code availability

All code and the trained segmentation model are publicly available on GitHub: https://github.com/OnofreyLab/volumetry-net

### Data

#### Inclusion of patients and magnetic resonance imaging data

From an institutional HCC database at Yale School of Medicine, all patients >18 years old were included that had T1-weighted portal venous MR images available for processing. All included scans were downloaded from the Picture Archiving and Communication System (PACS) server and de-identified. MRI was acquired between the years 2008 and 2019 using a standard triphasic institutional imaging protocol as suggested by the LIRADS comity [1]. T1-weighted 3D gradient echo volumetric interpolated examination (VIBE) sequence with fat saturation [18] were acquired before contrast administration and 12–18 s (depending on the bolus tracking), 60–70 s, and 3–5 min post-contrast injection for pre-contrast-, late arterial, portal venous-, and delayed-phase images, respectively, after the administration of various gadolinium-based contrast agents. The imaging was conducted using a range of scanners with different field strengths, including 1.16 T, 1.5 T, and 3 T. More information about imaging parameters can be found in Supplemental Table 1. 

#### External validation data

To evaluate the model’s generalization performance on a different patient population, an external dataset of de-identified T1-weighted portal venous MR images was made available from the Beaujon Hospital in Clichy, France. MRI was acquired between the years 2015 and 2020 using a standard triphasic institutional imaging protocol.

#### Public validation data

For additional external validation, the publicly available LiverHccSeg [19] dataset was used.

#### Image processing and liver segmentation

All images were converted to the Neuroimaging Informatics Technology Initiative (NIfTI) format. Subsequently, all livers were manually segmented under the supervision of two board-certified abdominal radiologists (S.A. and S.H. with 9 and 10 years of experience, respectively) using the software 3D Slicer (v4.11) [20].

### Model development

For model development and evaluation, our institutional dataset was randomly split into training, validation, and internal testing subsets containing 70/15/15% of the data, respectively.

A DCNN was trained to automatically segment the liver on 3D T1-weighted portal venous MR images using manual liver segmentations as ground truth. The architecture of the DCNN is based on the U-Net [21], including two residual units, and was trained with a dropout rate of 0.3, using mini-batches of size 32 with batch-normalization. To avoid model overfitting, we continuously assessed the performance of the model on the validation set during training over 1000 epochs to determine the best performing model. Full details of the model architecture can be found in Supplement 1. Input images were resized to have 2×2×2 mm voxel spacings and normalized such that the 25^th^ and 75^th^ image intensity percentiles were scaled to –0.5 and +0.5, respectively [22]. During every training iteration, 16 random 3D patches (64×64×32 voxels) were extracted from the input image in a 3:1 liver region-to-background ratio to focus the training process on the liver area. Dice loss [23] was optimized using the Adam optimizer [24] with a fixed learning rate of 0.0001. The resulting model comprised 1,187,921 trainable parameters and was implemented in Python (v3.8.3) using the open-source Medical Open Network for AI (MONAI) (v0.3.0) framework and PyTorch (v1.5.1) on a Linux workstation using an NVIDIA Quadro RTX 8000 GPU.

For model inference, a sliding window approach is used to segment the entire image field-of-view. Therefore, overlapping 3D patches of size 64×64×32 voxels were extracted at regular increments of 16×16×8 voxels. The prediction results of the overlapping patches were averaged using a Gaussian weighting according to the patch center. This approach accommodates images of different field-of-view sizes.

### Model evaluation and statistical analysis

#### Segmentation performance

To quantify segmentation accuracy, Dice similarity coefficient (DSC), Modified Hausdorff distance (MHD), and mean absolute distance (MAD) metrics were calculated between the manual and automated liver segmentations. The equations for calculating DSC, MHD, and MAD can be found in Supplement 2.

#### Volumetry performance

To assess liver volumetry accuracy, liver volumes were calculated based on the manual and automated liver segmentations. The intraclass correlation coefficient (ICC) (two-way mixed, single measures, ICC(3,1)) was calculated to assess the agreement between manual and automated volumetry. Furthermore, the absolute volume error and relative volume error were calculated as follows:

$$Absolute\ volume\ error=\left|{vol}_{man}-{vol}_{auto}\right|,$$

$$Relative\ volume\ error=\frac{\left|{vol}_{man}-{vol}_{auto}\right|}{vol_{man}},$$

where *vol*_*man*_ and *vol*_*auto*_ are the volumes from the manual and automated liver segmentations, respectively.

#### Radiomic feature reproducibility

A total of 107 radiomic features comprising 18 first-order statistic features, 24 gray-level co-occurrence matrix (glcm) features, 14 gray-level dependence matrix (gldm) features, 16 gray-level run-length matrix (glrlm) features, 16 gray-level size zone matrix (glszm) features, 5 neighboring gray-tone difference matrix (ngtdm) features, and 14 shape features were extracted from the manual and automated liver segmentations using the software PyRadiomics (v3.0) [25] and their agreement was assessed using the ICC(3,1). The PyRadiomics settings used for feature extraction are provided in Supplement 3.

#### Statistical analysis

Descriptive statistics were summarized as absolute and relative frequencies (*n* and %) for categorical variables or mean and standard deviation (SD) or median and interquartile range (IQR) for continuous variables. To assess data consistency across the training, validation, and internal testing sets, we employed a one-way ANOVA test for continuous characteristics, and a Chi-squared test for categorical characteristics. To evaluate the algorithm’s segmentation and volumetry generalizability, a Mann-Whitney *U* test was used to compare the segmentation performance from the internal to the external and public test sets, and between HCC and hepatic adenoma patients in the external test set. All statistical analyses were carried out in Python (v3.8.3) using the SciPy (v1.5.2) library and *p* values <0.05 were considered statistically significant.

## Results

### Internal study population

**Fig. 1:**
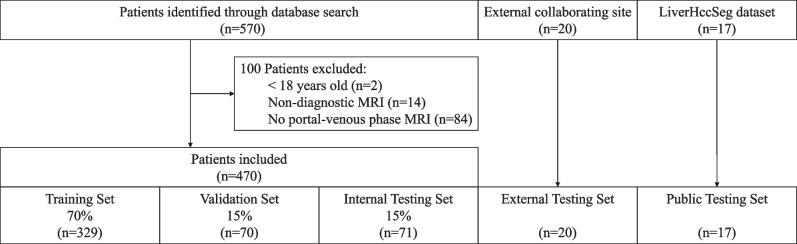
Flowchart of patient inclusion

A total of 470 HCC patients with T1-weighted portal venous MR images were included in this study (mean age, 63.9 years ± 8.2 [standard deviation]; 376 males). Patients <18 years (*n*=2), with non-diagnostic MRI (*n*=14), and no portal venous phase MRI (*n*=84) were excluded from the study (Fig. 1). Patient characteristics are summarized in Table 1, and MR imaging parameters are reported in Supplemental Table 1. HCC was either proven by imaging criteria or histopathology. Manual segmentation determined reference liver volumes (mean volume, 1687.9 ccm ± 534.7).

**Table 1:** Patient characteristics

Characteristic	Overall	Training set	Validation set	Internal test set	*p value*
Number of patients	470	329	70	71	
Mean age (years)	63.9 ± 8.2	63.9 ± 8.4	63.8 ± 8.2	64.0 ± 7.9	*0.99*^*†*^
Sex					*0.42*^*‡*^
Female	94 (20.0)	71 (21.6)	11 (15.7)	12 (16.9)	
Male	376 (80.0)	258 (78.4)	59 (84.3)	59 (83.1)	
Ethnicity*					*0.27*^*‡*^
Asian	13 (2.8)	12 (3.6)	1 (1.4)	0 (0)	
Black	57 (12.1)	40 (12.2)	9 (12.9)	8 (11.3)	
Hispanic	73 (15.5)	57 (17.3)	7 (10.0)	9 (12.7)	
Unknown	8 (1.7)	4 (1.2)	3 (4.3)	1 (1.4)	
White	319 (67.9)	216 (65.7)	50 (71.4)	53 (74.6)	
Cirrhosis	459 (97.7)	323 (98.2)	67 (95.7)	69 (97.2)	*0.45*^*‡*^
Etiology					
HCV*	281 (61.1)	202 (62.7)	37 (54.4)	42 (60.0)	*0.43*^*‡*^
HBV*	22 (4.9)	17 (5.4)	3 (4.6)	2 (3.0)	*0.74*^*‡*^
Alcohol	147 (32.4)	101 (31.4)	23 (35.4)	23 (34.3)	*0.77*^*‡*^
NAFLD*	75 (16.6)	44 (14.0)	13 (19.4)	18 (26.1)	*0.40*^*‡*^
Autoimmune	6 (1.3)	5 (1.6)	0 (0)	1 (1.5)	*0.60*^*‡*^
Cryptogenic	7 (1.6)	3 (1.0)	3 (4.6)	1 (1.5)	*0.96*^*‡*^
Not available	6 (1.3)	6 (1.9)	0 (0)	0 (0)	*0.28*^*‡*^
Radiological data					
Mean liver volume (ccm)	1687.9 ± 534.7	1668.0 ± 526.1	1715.9 ± 519.5	1752.2 ± 587.9	*0.43*^*†*^
Number of lesions					*0.96*^*‡*^
1	309 (65.7)	218 (66.3)	45 (64.3)	46 (64.8)	
2	93 (19.8)	64 (19.5)	14 (20.0)	15 (21.1)	
3	37 (7.9)	26 (7.9)	7 (10.0)	4 (5.6)	
>3	31 (6.6)	21 (6.4)	4 (5.7)	6 (8.5)	
Mean maximum tumor diameter (cm)	3.3 ± 2.0	3.3 ± 1.9	3.2 ± 1.7	3.3 ± 2.3	*0.96*^*†*^
Mean cumulative tumor diameter (cm)	3.9 ± 2.4	4.0 ± 2.4	3.8 ± 2.4	3.6 ± 2.2	*0.52*^*†*^
Liver lobe					*0.52*^*‡*^
Left	97 (20.6)	61 (18.5)	18 (25.7)	18 (25.4)	
Right	286 (60.9)	207 (62.9)	40 (57.1)	39 (54.9)	
Bilobar	87 (18.5)	61 (18.5)	12 (17.1)	14 (19.7)	
Ascites on imaging					*0.17*^*‡*^
Absent	361 (76.8)	263 (79.9)	48 (68.6)	50 (70.4)	
Mild	73 (15.5)	41 (12.5)	19 (27.1)	13 (18.3)	
Moderate	36 (7.7)	25 (7.6)	3 (4.3)	8 (11.3)	
Portal vein thrombosis	44 (9.4)	32 (9.7)	4 (5.7)	8 (11.3)	*0.48*^*‡*^
Tumor thrombus	29 (6.2)	19 (5.8)	4 (5.7)	6 (8.5)	*0.69*^*‡*^
Infiltrative disease	15 (3.2)	10 (3.0)	2 (2.9)	3 (4.2)	*0.86*^*‡*^

*Note. — *Numbers in parentheses are percentages. Ethnicity is provided through the electronic health record. To assess data consistency, datasets were compared using ^†^one-way ANOVA tests for continuous, and ^‡^Chi-squared tests for categorical characteristics

*HCV*, hepatitis C virus; *HBV*, hepatitis B virus; *NAFLD*, non-alcoholic fatty liver disease

### External study population

For external validation, 20 T1-weighted portal venous MR images were made available for external testing from a collaborating institution. Patients (mean age, 41 years ± 12; 13 males) were diagnosed with either HCC (*n*=10) or hepatic adenoma (*n*=10) and diagnoses were based on histopathology. Manual segmentation determined reference liver volumes (mean volume, 1728.4 ccm ± 647.7).

### Public study population

A total of 17 HCC patients (mean age, 61 years ± 10.77; 11 males) with T1-weighted portal venous MR images were included for additional external testing. Manual segmentation determined reference liver volumes (mean volume, 1916.3 ccm ± 459.6).

### Final segmentation model

The total training time of the network was 6.35 hours. Evaluation using the validation set identified the best performing model at epoch 880 where the validation DSC reached 0.97. The training and validation loss curves can be found in Supplemental Figure 3.

Algorithm segmentation time demonstrated no substantial differences between the internal (mean time, 0.54 s ± 0.20) and external (mean time, 0.70 s ± 0.70) test sets (*p=*0.17) or internal and public test sets (mean time, 0.604 s ± 0.230) (*p*=0.08).

### Segmentation performance

Segmentation accuracy of the DCNN was similarly high in the internal (mean DSC, 0.968 ± 0.016) and external (mean DSC, 0.961 ± 0.032) test sets (*p=*0.28). Notably, in the external test set the segmentation performance was similarly high in HCC (mean DSC, 0.970 ± 0.009) and hepatic adenoma (mean DSC, 0.953 ± 0.044) patients (*p*=0.21). The DCNN demonstrated adequate segmentation performance in the public test set (mean DSC, 0.93 ± 0.03). However, the overall segmentation performance in the public dataset was significantly lower than in the internal (*p<*0.001), and external (*p=*0.004) test sets.

**Fig. 2:**
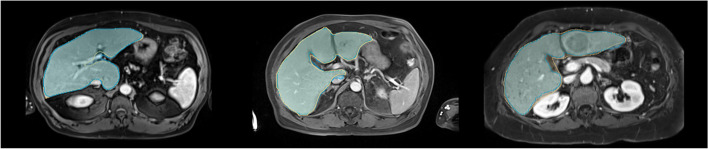
Representative segmentations. Representative liver segmentations on axial portal venous phase contrast-enhanced magnetic resonance images from the internal (left), external (middle), and public (right) test sets with Dice similarity coefficients of 0.977, 0.964, and 0.944, respectively. The manual liver segmentations are overlaid in yellow, and the automated segmentations are overlaid in blue

**Fig. 3:**
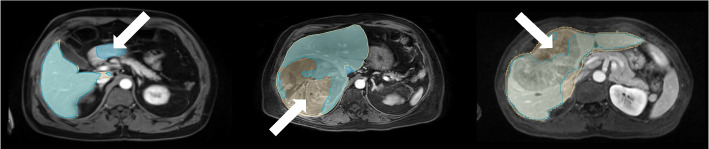
Segmentation failure cases. Liver segmentation failure cases from the internal (left), external (middle), and public (right) test sets with Dice similarity coefficients of 0.945, 0.830, and 0.889 respectively. Axial portal venous phase contrast-enhanced magnetic resonance images are shown with the corresponding manual liver segmentations overlaid in yellow, and the automated segmentations overlaid in blue. In the example on the left, the algorithm segmented parts of the duodenum as liver tissue (arrow). In the example in the middle and on the right, the algorithm excluded parts of a big tumor from the liver segmentation (arrows)

Table 2 summarizes all segmentation performance metrics for all datasets. Examples of representative liver segmentations are shown in Fig. 2, and Fig. 3 shows two segmentation failure cases with DSC <0.95 and poor qualitative performance. No substantial segmentation performance differences were noted across different clinical findings (Table 3; e.g., ascites, Supplemental Figure 1) or in cases with image artifacts or reduced image quality (Supplemental Figure 2) indicating robust generalizability.

**Table 2:** Liver segmentation performance

Performance metric	Mean	Standard deviation	Median	Interquartile range
DSC*				
Training	0.968	0.016	0.973	0.014
Validation	0.966	0.019	0.970	0.014
Internal testing	0.967	0.013	0.972	0.017
External testing	0.962	0.032	0.967	0.012
Public testing	0.928	0.031	0.932	0.027
MHD* (in voxels)				
Training	1.876	2.249	1.414	1.000
Validation	1.949	1.330	1.414	1.236
Internal testing	1.852	0.806	1.414	0.822
External testing	2.711	3.449	1.866	0.504
Public testing	6.893	6.790	3.452	5.969
MAD* (in voxels)				
Training	0.538	0.382	0.450	0.195
Validation	0.541	0.245	0.465	0.240
Internal testing	0.545	0.195	0.462	0.241
External testing	0.705	0.698	0.525	0.130
Public testing	1.625	1.371	1.138	0.644
Runtime (s)				
Training	0.538	0.382	0.450	0.195
Validation	0.541	0.245	0.465	0.240
Internal testing	0.545	0.195	0.462	0.241
External testing	0.705	0.698	0.525	0.130
Public testing	0.604	0.230	0.544	0.197

*Note. —* DSC, Dice Similarity Coefficient; MHD, Modified Hausdorff Distance; MAD, Mean Absolute Distance

**Table 3:** Statistical evaluation of performance generalizability

Parameter	Mean (±SD) DSC	*p* value^†^
Maximum tumor diameter		
<5 cm (*n*=56)	0.968 ± 0.012	*0.916*
≥5 cm (*n*=11)	0.966 ± 0.011	
Ascites		
Absent (*n*=50)	0.968 ± 0.013	*0.335*
Present (*n*=21)	0.965 ± 0.015	
Number of lesions		
1 (*n*=46)	0.967 ± 0.013	*0.351*
>1 (*n*=25)	0.968 ± 0.015	
Portal vein thrombosis		
Absent (*n*=63)	0.968 ± 0.013	*0.573*
Present (*n*=8)	0.963 ± 0.019	
Infiltrative disease		
Absent (*n*=68)	0.967 ± 0.014	*0.840*
Present (*n*=3)	0.971 ± 0.007	

*Note. —* ^†^Segmentation performance was compared between patient subgroups using a Mann-Whitney *U* test

*DSC* Dice similarity coefficient, *SD* standard deviation

### Volumetry performance

**Fig. 4:**
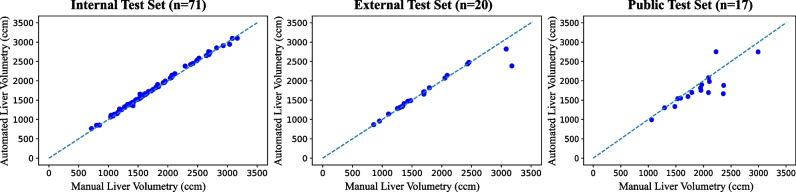
Scatter plots of liver volume measurements on portal venous phase contrast-enhanced magnetic resonance images determined by manual and automated liver volumetry. In the external test set, there were two cases in which the liver volumes were substantially underestimated corresponding to the cases with the lowest Dice similarity coefficients (0.830 and 0.949) resulting in absolute volume errors of 792.5 ccm and 260.9 ccm, respectively

Liver volumes determined by manual and automated liver volumetry demonstrated ICCs of 0.99 [95%CI 0.99, 1.00] (*p<*0.001), 0.97 [95%CI 0.93, 0.99] (*p<*0.001), and 0.85 [95%CI 0.62, 0.94] (*p<*0.001) for the internal, external, and public test sets, respectively. Absolute volume errors showed no statistical significance between the internal and external test sets (median volume, 31.7 ccm [interquartile range (IQR), 24.7] vs 19.3 ccm [IQR, 32.4]; *p*=0.12). Relative volume errors showed no statistical significance between the internal and external test sets (median error, 2.0% [IQR, 1.8] vs 1.3% [IQR, 2.0]; *p*=0.48). Table 4 summarizes the volumetry performance measures for all datasets. Comparisons of liver volumes determined by manual and automated volumetry can be found in the scatterplot in Fig. 4. In the external test set, there were two cases in which the algorithm substantially underestimated the liver volumes, corresponding to the cases with the lowest segmentation performance (DSC 0.830 and 0.949), resulting in absolute volume errors of 792.5 ccm and 260.9 ccm, respectively.

**Table 4:** Liver volumetry performance

Volumetry performance metric	Mean	Standard deviation	Median	Interquartile range
Absolute volume error (ccm)				
Training	34.3	24.1	30.1	16.9
Validation	33.6	16.6	30.1	18.3
Internal testing	38.8	22.6	31.7	24.7
External testing	72.2	178.3	19.3	32.4
Public testing	197.8	205.0	126.1	176.7
Relative volume error (% difference)				
Training	2.2	1.7	1.9	1.3
Validation	2.1	1.3	1.9	1.4
Internal testing	2.4	1.6	2.0	1.8
External testing	3.0	5.5	1.3	2.0
Public testing	9.4	8.6	6.9	6.7

### Radiomic feature reproducibility

Radiomic features derived from automated liver segmentations demonstrated significant agreement compared to manual segmentations (*p<*0.05; for all features in all datasets except the sphericity shape feature in the external test set: *p*=0.24) in the internal (mean ICC, 0.98 ± 0.04; range 0.80–1.00), external (mean ICC, 0.94 ± 0.10; range 0.16–1.00), and public (mean ICC, 0.91 ± 0.09; range 0.51–1.00) test sets. Supplemental Table 2 reports ICCs and 95% confidence intervals for each individual radiomic feature in each dataset.

## Discussion

Using a large dataset, we developed a deep learning algorithm for automated liver segmentation on T1-weighted portal venous phase contrast-enhanced MRI. Accurate liver segmentation is key for anatomical guidance in computer-assisted surgery [9] and radiotherapy [8, 10] and is also a pivotal pre-processing step for subsequent automated lesion detection algorithms [11]. Our segmentation framework attained robust liver segmentation results on internal (mean Dice similarity coefficient (DSC), 0.97), external (mean DSC, 0.96), and public (mean DSC, 0.93) test sets indicating generalizable performance to external data.

Radiological assessment of the liver is an important diagnostic task for hepatocellular carcinoma patients. Accurate and reproducible liver volumetry is key for clinical decision-making and treatment planning. Therefore, methods for automated volumetry enhance workflows and facilitate implementation into widespread clinical practice. Our method yielded fast processing times (mean runtime, 0.54 s) and automated liver volumetry demonstrated acceptable relative volume errors of 2.44% and 2.97%, and excellent agreement [26] with manual volumetry in the internal (intraclass correlation coefficient (ICC), 0.99) and external (ICC, 0.97) test sets and good agreement with manual volumetry in the public test set (ICC, 0.85). Finally, we demonstrate that automated liver segmentation provides robust and reproducible radiomic feature extraction compared to manual segmentation in the internal (mean ICC, 0.98), external (mean ICC, 0.94), and public (mean ICC, 0.91) test sets.

Traditional methods for liver segmentation are based on seeded region growing [27], support vector classification with watershed [28], watershed segmentation coupled with active contouring [29], and convolutional neural network with graph cut [30]. Each of them has their strengths and limitations, making them suitable for different scenarios. However, in comparison to traditional image segmentation methods, deep learning 3D convolutional neural networks offer superior performance, especially for complex and large-scale segmentation tasks when sufficient labeled training data is available. While traditional methods have the advantage of simplicity and computational efficiency for certain scenarios, DCNNs’ ability to learn intricate features from data has elevated their effectiveness in various medical imaging applications, including liver segmentation. It is worth noting that the success of DCNNs relies heavily on the availability of diverse labeled training data [16] and computational resources for training. Compared with other automated liver volumetry approaches, our approach showed higher volume agreement with higher ICC values and lower relative volume error rates [31–33]. Furthermore, external testing is critical when evaluating deep learning algorithms in order to avoid overfitting and dataset shift [14]. External test sets are infrequent and most studies report decreased model performance on external testing data [15], indicating limited generalizability. Only a small number of MRI liver segmentation studies [33, 34] evaluated algorithm performance on external testing data. In this study, we demonstrated generalizable performance by testing on a large internal test set (*n*=71), an external test set (*n*=20), and on the publicly available LiverHccSeg dataset (*n*=17) for further external validation.

Additionally, heterogeneous and diverse training data is key for generalizable and robust liver segmentation performance [16]. Advanced liver cancer substantially alters liver morphology as it leads to cancer-related tissue changes, heterogeneous liver tissue, and liver shape deformity [35, 36]. Deep learning methods not trained on data comprising the full spectrum of disease-related liver changes failed to segment livers with advanced tumor stages [16]. This contrasts with other methods evaluated on patient cohorts without intrahepatic lesions [34]. Other segmentation methods failed in patients with intrahepatic lesions that altered the liver parenchyma [33]. Our study utilized a diverse dataset of HCC patients with different disease etiologies (such as alcoholic steatohepatitis, non-alcoholic fatty liver disease, hepatitis C, hepatitis B, autoimmune, cryptogenic) that included a varying number of tumors of different sizes as well as portal vein thrombosis, tumor thrombi, infiltrative disease, and ascites. Furthermore, we used MRI scans acquired on a range of different scanners from different manufacturers with both extracellular and liver-specific contrast and some scans had limited image quality or artifacts as data encountered in real-world settings.

For the clinical implementation of automated liver segmentation and volumetry tools, simple processing routines and fast processing times are key for use in clinical practice. Our approach does not require extensive preprocessing, such as image registration, and takes less than 1 s of processing time, which compares favorably to other methods with longer processing times [31, 32, 37]. Since our method relies only on a single-contrast phase, image artifacts on any other imaging sequence do not change the quality of the liver segmentation and the resulting volume estimation. In comparison, methods using multiphasic data [37] are dependent on the image quality of all the used sequences and their registration. Other approaches performed liver segmentation on non-contrast MRI but obtained overall inferior DSC results [34, 38, 39].

Recently, quantitative imaging biomarkers such as radiomics have gained increased interest in liver disease studies [40] and automated segmentation methods are needed to reduce the segmentation effort required for feature extraction. Good reproducibility of radiomic features from automated segmentations was demonstrated in cervical cancer [41], but to our knowledge has not been confirmed in the liver. Here, we demonstrated feature robustness from the extracted radiomic features in the liver, which can be used for automated and reproducible radiomic feature extraction. While many studies use segmentations of specific regions or volumes of interest for feature extraction, whole-liver imaging biomarkers allow for a thorough assessment of the entire liver encompassing all its regions and structures [42]. This is particularly relevant in cases with multiple lesions or widespread liver disease. It reduces the risk of missing lesions in the segmentation and may be beneficial in cases with multiple or infiltrative lesions as they “blend” into the background of the cirrhotic liver [43] and therefore can be difficult to segment. Additionally, this automated liver segmentation approach could be used as pre-processing for other methods, such as deep learning, to extract novel imaging biomarkers from the liver beyond standard radiomics.

This study has limitations. First, the proposed automated liver segmentation method relies on contrast-enhanced T1-weighted MRI. However, in clinical practice, most scans are acquired using intravenous contrast for better detection and characterization of liver lesions. Second, this study only tested algorithm performance in patients with liver pathologies and requires further validation on healthy patients with healthy liver parenchyma. The observed discrepancy in model performance between the public dataset and our internal and external test sets could be attributed to several factors, primarily related to differences in the characteristics of the data. The public dataset, collected between 1993 and 2007, encompasses an earlier timeframe compared to our internal and external test sets. The advancements in MRI technology, imaging protocols, and the overall quality of medical imaging over the years may contribute to these disparities. Future work will assess the segmentation of liver lobes, liver segments, and liver vessels and we aim to expand our testing scope by incorporating larger test sets from multiple sites. Additionally, generalization to alternative MRI sequences, such as T2-weighted imaging, requires future validation. To this end, incremental learning or transfer learning strategies can leverage the model developed in this study as a starting point for future deep learning models that generalize across MRI sequences.

In conclusion, the presented deep convolutional neural network can perform accurate and fast liver segmentation on T1-weighted portal venous MR images with generalizable performance to external and public testing data. Automated liver volumetry shows excellent agreement with manual volumetry, and automated liver segmentations can be used for reliable radiomic feature extraction.

### Supplementary information

ESM 1 (DOCX 3.70 mb)

## Abbreviations

CI: Confidence interval
CNN: Convolutional neural network
DCNN: Deep convolutional neural network
DSC: Dice similarity coefficient
HCC: Hepatocellular carcinoma
ICC: Intraclass correlation coefficient
MAD: Mean absolute distance
MHD: Modified Hausdorff distance
MR: Magnetic resonance
MRI: Magnetic resonance imaging
SD: Standard deviation

## Declarations

### Guarantor

The scientific guarantor of this publication is Dr. John Onofrey.

### Statistics and biometry

No complex statistical methods were necessary for this paper.

### Study subjects or cohorts overlap

No study subjects or cohorts have been previously reported.

### Methodology

• retrospective

• experimental

• performed at one institution
