# Supplementary material for: Automated MRI liver segmentation for anatomical segmentation, liver volumetry, and the extraction of radiomics
Source: Eur Radiol. 2024 Jan 13;34(8):5056–65. doi: 10.1007/s00330-023-10495-5 (PMC11245591; doi:10.1007/s00330-023-10495-5)
Supplement: Supplementary file 1 — (DOCX 3.70 mb) [file 330_2023_10495_MOESM1_ESM.docx]

# Supplemental Material

for

**Automated MRI liver segmentation for anatomical segmentation, liver volumetry, and the extraction of imaging biomarkers**

**Supplemental Figure 1:** Axial portal-venous phase contrast-enhanced MRIs of HCC patients with ascites from the internal test set. The contours of the manual and automated liver segmentations are overlaid in orange and blue, respectively.

**Supplemental Figure 2:** Axial portal-venous phase contrast-enhanced MRIs of HCC patients with image artifacts and reduced image quality from the internal test set. The contours of the automated liver segmentations are overlaid in blue.

**Supplemental Figure 3:** The training and validation loss curves from the model development process. The validation loss was used to select the best performing model where the Dice Similarity Coefficient (DSC) loss had a minimum value at epoch 880.

**Supplemental Table 1:** Magnetic resonance imaging parameters.

| **Parameter** | **Overall** | **Training** | **Validation** | **Internal Testing** |
| --- | --- | --- | --- | --- |
| Number of scans | 470 | 329 | 70 | 71 |
| **Manufacturer** |  |  |  |  |
| GE Medical Systems | 98 (20.9) | 67 (20.4) | 18 (25.7) | 13 (18.3) |
| Hitachi | 5 (1.1) | 4 (1.2) | 0 (0.0) | 1 (1.4) |
| Philips | 9 (1.9) | 7 (2.1) | 1 (1.4) | 1 (1.4) |
| Siemens | 353 (75.1) | 249 (75.7) | 50 (71.4) | 54 (76.1) |
| Toshiba | 5 (1.1) | 2 (0.6) | 1 (1.4) | 2 (2.8) |
| **Model** |  |  |  |  |
| Achieva | 2 (0.4) | 2 (0.6) | 0 (0.0) | 0 (0.0) |
| Aera | 71 (15.1) | 42 (12.8) | 16 (22.9) | 13 (18.3) |
| Avanto | 81 (17.2) | 63 (19.1) | 10 (14.3) | 8 (11.3) |
| Discovery MR750w | 1 (0.2) | 1 (0.3) | 0 (0.0) | 0 (0.0) |
| Espree | 48 (10.2) | 32 (9.7) | 3 (4.3) | 13 (18.3) |
| Ingenia | 7 (1.5) | 5 (1.5) | 1 (1.4) | 1 (1.4) |
| MRT200PP3 | 1 (0.2) | 1 (0.3) | 0 (0.0) | 0 (0.0) |
| MRT200SP8 | 2 (0.4) | 0 (0.0) | 0 (0.0) | 2 (2.8) |
| OASIS | 5 (1.1) | 4 (1.2) | 0 (0.0) | 1 (1.4) |
| Optima MR450w | 8 (1.7) | 7 (2.1) | 1 (1.4) | 0 (0.0) |
| Signa Excite | 42 (8.9) | 27 (8.2) | 7 (10.0) | 8 (11.3) |
| Signa HDx | 27 (5.7) | 21 (6.4) | 5 (7.1) | 1 (1.4) |
| Signa HDxt | 15 (3.2) | 8 (2.4) | 3 (4.3) | 4 (5.6) |
| Skyra | 11 (2.3) | 6 (1.8) | 3 (4.3) | 2 (2.8) |
| Symphony | 8 (1.7) | 5 (1.5) | 2 (2.9) | 1 (1.4) |
| Symphony Tim | 1 (0.2) | 1 (0.3) | 0 (0.0) | 0 (0.0) |
| Trio Tim | 16 (3.4) | 11 (3.3) | 4 (5.7) | 1 (1.4) |
| Verio | 113 (24.0) | 86 (26.1) | 11 (15.7) | 16 (22.5) |
| Not available | 11 (2.3) | 7 (2.1) | 4 (5.7) | 0 (0.0) |
| **Magnetic Field Strength** |  |  |  |  |
| 1.16 T | 5 (1.1) | 4 (1.2) | 0 (0.0) | 1 (1.4) |
| 1.5 T | 308 (65.5) | 210 (63.8) | 49 (70.0) | 49 (69.0) |
| 3 T | 157 (33.4) | 115 (35.0) | 21 (30.0) | 21 (29.6) |
| **Contrast Agent Name** |  |  |  |  |
| Dotarem | 95 (20.2) | 61 (18.5) | 15 (21.4) | 19 (26.8) |
| Eovist | 24 (5.1) | 19 (5.8) | 4 (5.7) | 1 (1.4) |
| Gadovist | 234 (49.8) | 157 (47.7) | 38 (54.3) | 39 (54.9) |
| Magnevist | 90 (19.1) | 70 (21.3) | 11 (15.7) | 9 (12.7) |
| MultiHance | 14 (3.0) | 13 (4.0) | 1 (1.4) | 0 (0.0) |
| Omniscan | 4 (0.9) | 2 (0.6) | 0 (0.0) | 2 (2.8) |
| Optimark | 6 (1.3) | 5 (1.5) | 1 (1.4) | 0 (0.0) |
| ProHance | 3 (0.6) | 2 (0.6) | 0 (0.0) | 1 (1.4) |
| **Acquisition parameters** |  |  |  |  |
| Mean echo numbers | 1.0 ± 0.1 | 1.0 ± 0.1 | 1.0 ± 0.2 | 1.0 ± 0.1 |
| Mean echo time (ms) | 1.9 ± 0.5 | 1.9 ± 0.5 | 1.9 ± 0.5 | 1.9 ± 0.5 |
| Mean imaging frequency (MHz) | 83.6 ± 28.5 | 84.5 ± 28.8 | 81.8 ± 27.8 | 81.2 ± 27.7 |
| Mean bandwidth (Hz) | 410.7 ± 128.9 | 411.4 ± 126.5 | 413.6 ± 165.7 | 404.2 ± 95.4 |
| Mean repetition time (ms) | 4.4 ± 0.8 | 4.3 ± 0.8 | 4.4 ± 0.9 | 4.4 ± 0.9 |
| Mean slice thickness (mm) | 3.5 ± 0.9 | 3.5 ± 0.9 | 3.5 ± 0.9 | 3.5 ± 0.9 |
| Mean flip angle (°) | 10.3 ± 1.5 | 10.3 ± 1.6 | 10.3 ± 1.2 | 10.2 ± 1.0 |
| Mean echo train length | 2.0 ± 7.6 | 2.2 ± 8.5 | 1.1 ± 0.3 | 2.0 ± 7.3 |
| Echo spacing (ms) | 0 ± 0 | 0 ± 0 | 0 ± 0 | 0 ± 0 |
| ***Note. —*** Numbers in parentheses are percentages. | | | | |

**Supplemental Table 2:** Radiomic feature stability. Intraclass correlation coefficients (ICC) and 95% confidence intervals.

| **Feature name** | **Training** | **Validation** | **Internal testing** | **External testing** | **Public testing** |
| --- | --- | --- | --- | --- | --- |
| **First-order statistics (n=18)** | | |  |  |  |
| 10^th^ percentile | 1.0 [1.0 1.0] | 1.0 [1.0 1.0] | 1.0 [1.0 1.0] | 0.99 [0.99 1.0] | 0.94 [0.84 0.98] |
| 90^th^ percentile | 1.0 [1.0 1.0] | 1.0 [1.0 1.0] | 1.0 [1.0 1.0] | 0.88 [0.71.0 0.95] | 1.0 [0.99 1.0] |
| Energy | 1.0 [1.0 1.0] | 1.0 [1.0 1.0] | 1.0 [1.0 1.0] | 0.98 [0.95 0.99] | 1.0 [0.99 1.0] |
| Entropy | 1.0 [0.99 1.0] | 1.0 [1.0 1.0] | 1.0 [1.0 1.0] | 0.99 [0.97 0.99] | 0.93 [0.82 0.98] |
| Interquartile range | 1.0 [1.0 1.0] | 1.0 [1.0 1.0] | 1.0 [1.0 1.0] | 0.99 [0.97 1.0] | 0.96 [0.89 0.99] |
| Kurtosis | 0.99 [0.99 1.0] | 0.99 [0.99 1.0] | 1.0 [0.99 1.0] | 0.99 [0.97 1.0] | 0.9 [0.73 0.96] |
| Maximum | 0.93 [0.91.0 0.95] | 0.98 [0.97 0.99] | 0.93 [0.88 0.96] | 0.97 [0.93 0.99] | 0.96 [0.90 0.99] |
| Mean absolute deviation | 1.0 [0.99 1.0] | 1.0 [1.0 1.0] | 1.0 [1.0 1.0] | 0.98 [0.94 0.99] | 0.93 [0.81.0 0.97] |
| Mean | 1.0 [1.0 1.0] | 1.0 [1.0 1.0] | 1.0 [1.0 1.0] | 0.99 [0.98 1.0] | 0.99 [0.98 1.0] |
| Median | 1.0 [1.0 1.0] | 1.0 [1.0 1.0] | 1.0 [1.0 1.0] | 0.99 [0.99 1.0] | 0.99 [0.99 1.0] |
| Minimum | 0.91.0 [0.89 0.93] | 0.91.0 [0.84 0.95] | 0.89 [0.81.0 0.93] | 0.91.0 [0.79 0.97] | 0.7 [0.35 0.88] |
| Range | 0.93 [0.91.0 0.94] | 0.92 [0.87 0.96] | 0.89 [0.82 0.94] | 0.94 [0.86 0.98] | 0.68 [0.31.0 0.87] |
| Robust mean absolute deviation | 1.0 [1.0 1.0] | 1.0 [1.0 1.0] | 1.0 [1.0 1.0] | 0.99 [0.96 0.99] | 0.96 [0.89 0.98] |
| Root mean squared | 1.0 [1.0 1.0] | 1.0 [1.0 1.0] | 1.0 [1.0 1.0] | 0.99 [0.98 1.0] | 1.0 [0.99 1.0] |
| Skewness | 1.0 [1.0 1.0] | 1.0 [1.0 1.0] | 1.0 [1.0 1.0] | 0.99 [0.99 1.0] | 0.96 [0.90 0.99] |
| Total energy | 1.0 [1.0 1.0] | 1.0 [1.0 1.0] | 1.0 [1.0 1.0] | 0.98 [0.95 0.99] | 1.0 [0.99 1.0] |
| Uniformity | 1.0 [1.0 1.0] | 1.0 [1.0 1.0] | 1.0 [1.0 1.0] | 1.0 [0.99 1.0] | 0.97 [0.92 0.99] |
| Variance | 0.99 [0.99 0.99] | 1.0 [1.0 1.0] | 0.99 [0.99 1.0] | 0.95 [0.87 0.98] | 0.88 [0.70 0.96] |
| **Gray-level co-occurrence matrix (glcm) (n=24)** | | | |  |  |
| Autocorrelation | 0.95 [0.94 0.96] | 0.94 [0.90 0.97] | 0.95 [0.91.0 0.97] | 0.94 [0.86 0.98] | 0.86 [0.66 0.95] |
| Cluster prominence | 0.99 [0.99 0.99] | 1.0 [0.99 1.0] | 0.98 [0.96 0.99] | 0.86 [0.67 0.94] | 0.85 [0.64 0.94] |
| Cluster shade | 0.99 [0.99 1.0] | 1.0 [1.0 1.0] | 0.99 [0.99 1.0] | 0.95 [0.86 0.98] | 0.94 [0.84 0.98] |
| Cluster tendency | 0.99 [0.99 1.0] | 1.0 [1.0 1.0] | 1.0 [0.99 1.0] | 0.96 [0.9 0.98] | 0.9 [0.74 0.96] |
| Contrast | 0.99 [0.99 1.0] | 0.99 [0.98 1.0] | 0.99 [0.99 1.0] | 0.9 [0.77 0.96] | 0.95 [0.86 0.98] |
| Correlation | 1.0 [0.99 1.0] | 1.0 [1.0 1.0] | 1.0 [0.99 1.0] | 0.99 [0.99 1.0] | 0.96 [0.90 0.99] |
| Difference average | 1.0 [1.0 1.0] | 1.0 [0.99 1.0] | 1.0 [1.0 1.0] | 0.97 [0.92 0.99] | 0.98 [0.94 0.99] |
| Difference entropy | 1.0 [1.0 1.0] | 0.99 [0.99 1.0] | 1.0 [0.99 1.0] | 0.94 [0.85 0.98] | 0.98 [0.95 0.99] |
| Difference variance | 0.99 [0.99 0.99] | 0.99 [0.97 0.99] | 0.99 [0.98 0.99] | 0.81.0 [0.57 0.92] | 0.92 [0.79 0.97] |
| Inverse difference | 1.0 [1.0 1.0] | 1.0 [1.0 1.0] | 1.0 [1.0 1.0] | 0.98 [0.96 0.99] | 0.99 [0.97 1.0] |
| Inverse difference moment | 1.0 [1.0 1.0] | 1.0 [1.0 1.0] | 1.0 [1.0 1.0] | 0.98 [0.95 0.99] | 0.99 [0.97 1.0] |
| Inverse difference moment normalized | 0.98 [0.97 0.98] | 0.98 [0.96 0.99] | 0.95 [0.91.0 0.97] | 0.94 [0.86 0.98] | 0.93 [0.81.0 0.97] |
| Inverse difference normalized | 0.99 [0.99 0.99] | 0.99 [0.98 0.99] | 0.98 [0.96 0.99] | 0.98 [0.94 0.99] | 0.96 [0.90 0.99] |
| Informational measure of correlation 1 | 1.0 [1.0 1.0] | 1.0 [1.0 1.0] | 1.0 [1.0 1.0] | 1.0 [0.99 1.0] | 0.99 [0.96 0.99] |
| Informational measure of correlation 2 | 1.0 [1.0 1.0] | 1.0 [1.0 1.0] | 1.0 [1.0 1.0] | 1.0 [1.0 1.0] | 0.98 [0.95 0.99] |
| Inverse variance | 1.0 [1.0 1.0] | 1.0 [1.0 1.0] | 1.0 [1.0 1.0] | 0.99 [0.98 1.0] | 0.99 [0.96 0.99] |
| Joint average | 0.96 [0.95 0.97] | 0.95 [0.92 0.97] | 0.96 [0.92 0.97] | 0.94 [0.86 0.98] | 0.89 [0.73 0.96] |
| Joint energy | 1.0 [1.0 1.0] | 1.0 [1.0 1.0] | 1.0 [1.0 1.0] | 0.99 [0.99 1.0] | 0.96 [0.89 0.99] |
| Joint entropy | 1.0 [0.99 1.0] | 1.0 [1.0 1.0] | 1.0 [0.99 1.0] | 0.98 [0.94 0.99] | 0.95 [0.86 0.98] |
| Maximal correlation coefficient | 1.0 [1.0 1.0] | 1.0 [1.0 1.0] | 1.0 [0.99 1.0] | 0.99 [0.96 0.99] | 0.97 [0.93 0.99] |
| Maximum probability | 1.0 [1.0 1.0] | 1.0 [1.0 1.0] | 1.0 [1.0 1.0] | 1.0 [0.99 1.0] | 0.98 [0.94 0.99] |
| Sum average | 0.96 [0.95 0.97] | 0.95 [0.92 0.97] | 0.96 [0.92 0.97] | 0.94 [0.86 0.98] | 0.89 [0.73 0.96] |
| Sum entropy | 1.0 [1.0 1.0] | 1.0 [1.0 1.0] | 1.0 [1.0 1.0] | 0.99 [0.97 1.0] | 0.93 [0.82 0.97] |
| Sum squares | 0.99 [0.99 1.0] | 1.0 [1.0 1.0] | 1.0 [0.99 1.0] | 0.96 [0.89 0.98] | 0.89 [0.73 0.96] |
| **Gray level dependence matrix (gldm) (n=14)** | | | |  |  |
| Dependence entropy | 1.0 [1.0 1.0] | 1.0 [1.0 1.0] | 1.0 [1.0 1.0] | 0.99 [0.98 1.0] | 0.97 [0.92 0.99] |
| Dependence non uniformity | 1.0 [1.0 1.0] | 1.0 [1.0 1.0] | 1.0 [1.0 1.0] | 0.96 [0.9 0.99] | 0.9 [0.75 0.96] |
| Dependence non uniformity normalized | 1.0 [1.0 1.0] | 1.0 [1.0 1.0] | 1.0 [1.0 1.0] | 0.98 [0.95 0.99] | 0.99 [0.98 1.0] |
| Dependence variance | 1.0 [1.0 1.0] | 1.0 [1.0 1.0] | 1.0 [1.0 1.0] | 0.98 [0.95 0.99] | 1.0 [0.99 1.0] |
| Gray level non uniformity | 1.0 [1.0 1.0] | 1.0 [1.0 1.0] | 1.0 [1.0 1.0] | 1.0 [0.99 1.0] | 0.92 [0.80 0.97] |
| Gray level variance | 0.99 [0.99 0.99] | 1.0 [1.0 1.0] | 0.99 [0.99 1.0] | 0.95 [0.87 0.98] | 0.88 [0.70 0.96] |
| High gray level emphasis | 0.95 [0.94 0.96] | 0.94 [0.90 0.97] | 0.95 [0.91.0 0.97] | 0.94 [0.86 0.98] | 0.86 [0.65 0.95] |
| Large dependence emphasis | 1.0 [1.0 1.0] | 1.0 [1.0 1.0] | 1.0 [1.0 1.0] | 0.99 [0.98 1.0] | 0.99 [0.97 1.0] |
| Large dependence high gray level emphasis | 0.97 [0.96 0.97] | 0.96 [0.93 0.98] | 0.96 [0.93 0.98] | 0.97 [0.92 0.99] | 0.91.0 [0.78 0.97] |
| Large dependence low gray level emphasis | 0.98 [0.98 0.99] | 0.99 [0.98 0.99] | 0.98 [0.97 0.99] | 0.95 [0.88 0.98] | 0.98 [0.95 0.99] |
| Low gray level emphasis | 0.96 [0.95 0.97] | 0.98 [0.96 0.99] | 0.96 [0.93 0.98] | 0.93 [0.84 0.97] | 0.89 [0.72 0.96] |
| Small dependence emphasis | 1.0 [1.0 1.0] | 0.99 [0.99 1.0] | 1.0 [0.99 1.0] | 0.95 [0.87 0.98] | 0.99 [0.96 0.99] |
| Small dependence high gray level emphasis | 0.98 [0.97 0.98] | 0.97 [0.95 0.98] | 0.97 [0.96 0.99] | 0.92 [0.81.0 0.97] | 0.95 [0.87 0.98] |
| Small dependence low gray level emphasis | 0.84 [0.80 0.87] | 0.9 [0.83 0.94] | 0.85 [0.75 0.91] | 0.64 [0.28 0.85] | 0.51.0 [0.05 0.79] |
| **Gray level run length matrix (glrlm) (n=16)** | | | |  |  |
| Gray level non uniformity | 1.0 [1.0 1.0] | 1.0 [0.99 1.0] | 1.0 [1.0 1.0] | 0.99 [0.98 1.0] | 0.9 [0.76 0.96] |
| Gray level non uniformity normalized | 1.0 [0.99 1.0] | 1.0 [1.0 1.0] | 1.0 [0.99 1.0] | 0.99 [0.97 1.0] | 0.97 [0.91.0 0.99] |
| Gray level variance | 0.99 [0.99 0.99] | 1.0 [0.99 1.0] | 0.99 [0.98 1.0] | 0.89 [0.74 0.96] | 0.86 [0.66 0.95] |
| High gray level run emphasis | 0.95 [0.93 0.96] | 0.93 [0.89 0.96] | 0.94 [0.89 0.96] | 0.94 [0.84 0.97] | 0.85 [0.63 0.94] |
| Long run emphasis | 1.0 [1.0 1.0] | 1.0 [1.0 1.0] | 1.0 [1.0 1.0] | 0.99 [0.98 1.0] | 0.99 [0.96 0.99] |
| Long run high gray level emphasis | 0.98 [0.98 0.99] | 0.98 [0.96 0.99] | 0.98 [0.97 0.99] | 0.98 [0.96 0.99] | 0.82 [0.58 0.93] |
| Long run low gray level emphasis | 0.99 [0.99 0.99] | 0.99 [0.99 1.0] | 0.99 [0.99 1.0] | 0.96 [0.9 0.98] | 0.95 [0.88 0.98] |
| Low gray level run emphasis | 0.95 [0.94 0.96] | 0.96 [0.93 0.98] | 0.95 [0.91.0 0.97] | 0.88 [0.71.0 0.95] | 0.88 [0.69 0.95] |
| Run entropy | 1.0 [1.0 1.0] | 1.0 [1.0 1.0] | 1.0 [1.0 1.0] | 1.0 [0.99 1.0] | 0.94 [0.85 0.98] |
| Run length non uniformity | 1.0 [1.0 1.0] | 0.99 [0.99 1.0] | 1.0 [1.0 1.0] | 0.93 [0.83 0.97] | 0.84 [0.61.0 0.94] |
| Run length non uniformity normalized | 1.0 [1.0 1.0] | 1.0 [1.0 1.0] | 1.0 [1.0 1.0] | 0.96 [0.91.0 0.99] | 0.99 [0.96 1.0] |
| Run percentage | 1.0 [1.0 1.0] | 1.0 [1.0 1.0] | 1.0 [1.0 1.0] | 0.99 [0.97 1.0] | 0.99 [0.97 1.0] |
| Run variance | 1.0 [1.0 1.0] | 1.0 [1.0 1.0] | 1.0 [1.0 1.0] | 0.99 [0.98 1.0] | 0.99 [0.97 1.0] |
| Short run emphasis | 1.0 [1.0 1.0] | 1.0 [0.99 1.0] | 1.0 [1.0 1.0] | 0.96 [0.89 0.98] | 0.98 [0.96 0.99] |
| Short run high gray level emphasis | 0.96 [0.95 0.97] | 0.95 [0.91.0 0.97] | 0.95 [0.92 0.97] | 0.93 [0.82 0.97] | 0.87 [0.67 0.95] |
| Short run low gray level emphasis | 0.93 [0.91.0 0.94] | 0.93 [0.88 0.96] | 0.91.0 [0.84 0.95] | 0.79 [0.54 0.91] | 0.84 [0.62 0.94] |
| **Gray level size zone matrix (glszm) (n=16)** | | | |  |  |
| Gray level non uniformity | 0.99 [0.99 1.0] | 0.99 [0.98 0.99] | 0.99 [0.98 0.99] | 0.98 [0.94 0.99] | 0.92 [0.78 0.97] |
| Gray level non uniformity normalized | 0.97 [0.97 0.98] | 0.97 [0.95 0.98] | 0.98 [0.96 0.99] | 0.89 [0.75 0.96] | 0.88 [0.71.0 0.96] |
| Gray level variance | 0.97 [0.96 0.98] | 0.98 [0.97 0.99] | 0.98 [0.96 0.99] | 0.83 [0.61.0 0.93] | 0.86 [0.65 0.95] |
| High gray level zone emphasis | 0.9 [0.87 0.92] | 0.89 [0.81.0 0.93] | 0.87 [0.78 0.92] | 0.83 [0.61.0 0.93] | 0.78 [0.50 0.92] |
| Large area emphasis | 1.0 [1.0 1.0] | 1.0 [1.0 1.0] | 1.0 [1.0 1.0] | 0.98 [0.94 0.99] | 0.99 [0.97 1.0] |
| Large area high gray level emphasis | 0.98 [0.98 0.99] | 0.98 [0.97 0.99] | 0.98 [0.97 0.99] | 0.99 [0.99 1.0] | 0.92 [0.8 0.97] |
| Large area low gray level emphasis | 0.99 [0.98 0.99] | 0.99 [0.99 1.0] | 1.0 [1.0 1.0] | 0.92 [0.82 0.97] | 0.98 [0.93 0.99] |
| Low gray level zone emphasis | 0.82 [0.77 0.86] | 0.83 [0.73 0.9 ] | 0.82 [0.70 0.89] | 0.67 [0.33 0.86] | 0.81.0 [0.54 0.93] |
| Size zone non uniformity | 0.99 [0.99 1.0] | 0.99 [0.98 0.99] | 0.99 [0.99 1.0] | 0.94 [0.85 0.98] | 0.92 [0.80 0.97] |
| Size zone non uniformity normalized | 0.99 [0.99 0.99] | 0.99 [0.98 0.99] | 0.99 [0.99 1.0] | 0.93 [0.82 0.97] | 0.97 [0.93 0.99] |
| Small area emphasis | 0.99 [0.99 0.99] | 0.99 [0.98 0.99] | 0.99 [0.98 0.99] | 0.91.0 [0.79 0.97] | 0.97 [0.93 0.99] |
| Small area high gray level emphasis | 0.93 [0.91.0 0.95] | 0.91.0 [0.86 0.95] | 0.92 [0.87 0.96] | 0.83 [0.6 0.93] | 0.81.0 [0.55 0.93] |
| Small area low gray level emphasis | 0.77 [0.71.0 0.82] | 0.79 [0.66 0.87] | 0.8 [0.67 0.88] | 0.71.0 [0.39 0.88] | 0.73 [0.40 0.89] |
| Zone entropy | 0.99 [0.98 0.99] | 0.99 [0.98 0.99] | 0.99 [0.98 0.99] | 0.96 [0.9 0.98] | 0.97 [0.91.0 0.99] |
| Zone percentage | 1.0 [0.99 1.0] | 0.99 [0.99 1.0] | 1.0 [0.99 1.0] | 0.97 [0.92 0.99] | 0.98 [0.95 0.99] |
| Zone variance | 1.0 [1.0 1.0] | 1.0 [1.0 1.0] | 1.0 [1.0 1.0] | 0.98 [0.94 0.99] | 0.99 [0.97 1.0] |
| **Neighboring gray tone difference matrix (ngtdm) (n=5)** | | | | |  |
| Busyness | 0.95 [0.94 0.96] | 0.98 [0.96 0.99] | 0.97 [0.94 0.98] | 0.91.0 [0.78 0.96] | 0.83 [0.59 0.93] |
| Coarseness | 1.0 [1.0 1.0] | 1.0 [1.0 1.0] | 1.0 [1.0 1.0] | 0.99 [0.99 1.0] | 0.94 [0.83 0.98] |
| Complexity | 0.96 [0.95 0.97] | 0.95 [0.91.0 0.97] | 0.95 [0.91.0 0.97] | 0.95 [0.87 0.98] | 0.93 [0.81.0 0.97] |
| Contrast | 0.98 [0.97 0.98] | 0.99 [0.99 1.0] | 0.98 [0.97 0.99] | 0.94 [0.85 0.98] | 0.88 [0.69 0.95] |
| Strength | 0.95 [0.94 0.96] | 0.94 [0.90 0.97] | 0.93 [0.87 0.96] | 0.91.0 [0.78 0.96] | 0.67 [0.29 0.87] |
| **Shape (n=14)** | |  |  |  |  |
| Elongation | 0.99 [0.99 1.0] | 1.0 [1.0 1.0] | 0.99 [0.99 1.0] | 0.99 [0.97 1.0] | 0.96 [0.90 0.99] |
| Flatness | 1.0 [0.99 1.0] | 1.0 [1.0 1.0] | 1.0 [0.99 1.0] | 0.99 [0.97 1.0] | 0.94 [0.85 0.98] |
| Least axis length | 1.0 [0.99 1.0] | 1.0 [1.0 1.0] | 1.0 [1.0 1.0] | 1.0 [0.99 1.0] | 0.89 [0.71.0 0.96] |
| Major axis length | 0.99 [0.99 1.0] | 0.99 [0.99 1.0] | 0.99 [0.99 1.0] | 0.99 [0.97 1.0] | 0.96 [0.90 0.99] |
| Maximum 2D diameter coronal | 0.97 [0.96 0.98] | 0.98 [0.96 0.99] | 0.97 [0.96 0.99] | 0.94 [0.85 0.98] | 0.91.0 [0.77 0.97] |
| Maximum 2D diameter axial | 0.99 [0.99 0.99] | 0.98 [0.97 0.99] | 0.98 [0.97 0.99] | 0.99 [0.98 1.0] | 0.88 [0.69 0.95] |
| Maximum 2D diameter slice | 0.99 [0.98 0.99] | 1.0 [0.99 1.0] | 0.99 [0.99 1.0] | 0.98 [0.96 0.99] | 0.92 [0.80 0.97] |
| Maximum 3D diameter | 0.98 [0.98 0.99] | 0.99 [0.99 1.0] | 0.99 [0.98 0.99] | 0.96 [0.89 0.98] | 0.87 [0.69 0.95] |
| Mesh volume | 1.0 [1.0 1.0] | 1.0 [1.0 1.0] | 1.0 [1.0 1.0] | 0.95 [0.87 0.98] | 0.85 [0.63 0.94] |
| Minor axis length | 1.0 [1.0 1.0] | 0.99 [0.99 1.0] | 1.0 [0.99 1.0] | 1.0 [0.99 1.0] | 0.96 [0.90 0.99] |
| Sphericity | 0.94 [0.93 0.96] | 0.97 [0.94 0.98] | 0.96 [0.94 0.98] | 0.16 [-0.3 0.57] | 0.60 [0.19 0.84] |
| Surface area | 0.99 [0.99 0.99] | 0.99 [0.99 1.0] | 1.0 [0.99 1.0] | 0.97 [0.92 0.99] | 0.85 [0.63 0.94] |
| Surface volume ratio | 0.99 [0.99 0.99] | 0.99 [0.98 0.99] | 0.99 [0.99 1.0] | 0.71.0 [0.39 0.88] | 0.79 [0.51.0 0.92] |
| Voxel volume | 1.0 [1.0 1.0] | 1.0 [1.0 1.0] | 1.0 [1.0 1.0] | 0.95 [0.87 0.98] | 0.85 [0.63 0.94] |
| ***Note.*** *—* Radiomic features were extracted from manual and automated liver segmentations using the software PyRadiomics (1). Equations for each feature can be found in ref. (2). To evaluate reproducibility of the extracted radiomic features, the intraclass correlation coefficient (ICC) (two-way mixed, single measures, ICC(3,1)) and 95% confidence intervals (CI) were calculated between the manual and automated segmentation method. | | | | | |

**Supplement 1:** Deep learning model architecture.

An adopted 3D U-net architecture was used and implemented in the open-source Medical Open Network for AI (MONAI) (v0.3.0) framework (3). This network was parameterized to have encoding blocks with the following components: a convolution layer of stride 2 to downsample the data followed by a residual unit consisting of two units of convolution, normalization (Batch Norm), dropout, and activation (PReLU) repeated in succession. The parameterization uses four encoding blocks with 16, 32, 64, and 128 features, respectively. The decoding blocks take the form: a convolution transpose layer to upsample the data followed by Batch Norm, dropout, and PReLU layers and then a residual unit consisting of convolution, Batch Norm, dropout, and PReLU. All convolution operations use 3x3x3 kernels, and the convolution transpose operations use 2x2x2 kernels with stride size 2. The final segmentation convolution layer uses a 1x1x1 kernel with stride 1 and has 2 features. In total, the model contains 1,187,921 trainable parameters. Model weights were initialized using the default PyTorch method to have random weights uniformly sampled between [-*k*,+*k*] where $k=\sqrt{1/kernel size}$. No data augmentation was used. To remove segmentation artifacts, all results were post-processed to keep only the largest connected component (the estimated liver mask) in the image. Full network details can be found in the source code repository (https://github.com/OnofreyLab/volumetry-net).

**Supplement 2:** Definition of segmentation performance metrics.

The following segmentation metrics were calculated: Dice Similarity Coefficient (DSC), Modified Hausdorff Distance (MHD), and Mean Absolute Distance (MAD).

DSC measures the overlap between the automated and the manual liver segmentation:

$$DSC(A,B)=\frac{2A\cap B}{A+B}$$

Where A and B are the automated and manual segmentations, respectively.

To calculate MHD and MAD, the liver segmentation surfaces A and B were extracted.

Hausdorff Distance (HD) is calculated as:

$$HD(A,B)=max\left\{ \max_{a\in A} d(a,B),\left. \max_{b\in B} d(b,A) \right\} \right.$$

where $d(a,B)= {min}_{b \in B}\left\| b-a \right\|^{2}$.

MAD was calculated as:

$$MAD(A,B)=\frac{1}{2}\left\{ \frac{1}{N_{A}}\sum_{a\in A} d(a,B)+\frac{1}{N_{B}}\sum_{b\in B} d(b,A) \right\}$$

**Supplement 3:** PyRadiomics feature extraction settings.

Radiomic features were extracted from the manual and automated liver segmentations using the software PyRadiomics (v3.0) (1) with the following extraction parameters:

imageType:

Original:

setting:

additionalInfo: True,

binWidth: 25,

interpolator: sitkBSpline,

label: 1,

normalize: True,

normalizeScale: 100,

removeOutliers: 3,

resampledPixelSpacing:

- 1.25

- 1.25

- 1.25

**Supplemental Material References**

1. van Griethuysen JJM, Fedorov A, Parmar C, Hosny A, Aucoin N, Narayan V, Beets-Tan RGH, Fillion-Robin JC, Pieper S, Aerts H. Computational Radiomics System to Decode the Radiographic Phenotype. Cancer Res 2017;77(21):e104-e107. doi: 10.1158/0008-5472.CAN-17-0339

2. Pyradiomics-community. pyradiomics Documentation Release v3.0. https://pyradiomics.readthedocs.io/en/v3.0/features.html. Published February 11, 2020. Accessed February 24, 2023.

3. Kerfoot E, Clough J, Oksuz I, Lee J, King AP, Schnabel JA. Left-Ventricle Quantification Using Residual U-Net. Statistical Atlases and Computational Models of the Heart Atrial Segmentation and LV Quantification Challenges STACOM 2018 Lecture Notes in Computer Science 2019;11395:371-380. doi: 10.1007/978-3-030-12029-0_40
